# *IJHPR* comes of age

**DOI:** 10.1186/2045-4015-3-45

**Published:** 2014-12-23

**Authors:** Bruce Rosen, Avi Israeli

**Affiliations:** Myers-JDC-Brookdale Institute, POB 3886, Jerusalem, 91037 Israel; Hadassah–Hebrew University Medical Center, POB 12000, Jerusalem, 91120 Israel; Chief Scientist’s Office, Ministry of Health, Ben Tabai 2, Jerusalem, 93591 Israel

## Abstract

The *Israel Journal of Health Policy Research (IJHPR)* is about to complete its third year of publication. Over the past year, the journal received its first impact factor, three of its articles crossed the 10,000 accesses threshold, submissions increased by 50%, and the journal published its fifth article collection. These accomplishments are due to the combined efforts of the National Institute of Health Policy and its leadership team, *IJHPR's* editorial board, our publisher BioMed Central, and the journal's authors, commentators and reviewers from Israel and around the world.

In Jewish tradition, the transition from a new endeavor's third year to its fourth year is full of significance, reflecting a phase transition in terms of maturity and stability. After 3 years of publication, *IJHPR* is also in the process of moving into a new phase; a more mature phase, with greater stability and a firmer place in the professional literature. We hope that this stability can be used as a springboard for further innovation, experimentation and growth.

With the *Israel Journal of Health Policy Research (IJHPR)* about to complete its third year of publication, we are pleased to have this opportunity to share some reflections about the year gone by and some of our hopes for the year – and years - ahead.

One of *IJHPR's* most significant achievements during the course of 2014 was that, in July, the journal received its first impact factor from Thomson Reuters. Perhaps more significant than the actual number (1.25) was the speed with which the journal received its first impact factor; it is extremely rare for this to happen during a journal's first three years of publication. Our understanding is that this achievement is due, first and foremost, to the journal's consistency in publishing a steady stream of high quality articles, on a broad range of significant health policy issues. Other important factors were the journal's unique niche, as a journal combining a national focus with international perspectives, and the high production standards maintained by our publisher, BioMed Central.

Another important achievement is that the journal's articles are reaching a wide and diverse audience. Three of our articles [1–3] have now been accessed at least 10,000 times, twelve have been accessed at least 5,000 times, and 75 have been accessed at least 2,000 times.^a^ Over 60% of the article accesses are from countries other than Israel. Naturally enough, most of these are from the English-speaking countries – USA, UK, Canada, and Australia. But we are also honored to have large numbers of readers from such countries as India, Brazil and even Indonesia.

We are also very pleased by another aspect of the journal's diversity – the range of institutions represented among the authors of the journal's articles. Virtually all of Israel's major institutions are well represented, and several prestigious institutions from outside of Israel are also associated with multiple *IJHPR* articles.

In 2014, the journal also experienced a major up-tick in the number of submissions; as of the end of November, we are running 50% above the year-to-date number for 2013 and 100% above the year-to-date number for 2012. The receipt of an impact factor in July, apparently played a major role in encouraging more submissions. It may be that word-of-mouth encouragement from authors who have been pleased with the quality and courtesy of *IJHPR's* review process has also played a role.

To date, *IJHPR* has published 140 articles and, as most articles have more than one author, the number of authors who have published with us is substantially greater – approximately 350. We are greatly appreciative of all of those colleagues who have entrusted us with their hard-earned manuscripts, and hope that we have lived up to their expectations. We are particularly appreciative of those colleagues who submitted manuscripts to *IJHPR* even before it got "on the map" this past July. We look forward to additional submissions from colleagues who have already published in *IJHPR* and to expanding the circle of contributors to those who have not yet published with us.

It is also noteworthy that in 2014 the journal published its fifth article collection. It now has collections on the following topics – quality, equity, prioritization, health promotion & disease prevention, and the healthcare workforce. These collections continue to grow; we are pleased to have heard that they (as well as individual *IJHPR* articles) increasingly serve as unique resources for both policy analysts and for scholars engaged in research or teaching about Israeli health care. Links to all these collections can be found at: http://www.ijhpr.org/series.

Our publisher, BioMed Central, has indicated that it is delighted with *IJHPR’s* progress and the quality of its publications. It feels that the accomplishments to date bode well for the long term future of the journal.

As we look forward to the year, and years, ahead, we hope to build on these achievements and to introduce various new features and modes of operation so that we can handle the growing volume of submissions without sacrificing either speed or quality. For example, we are considering publishing special issues on topics of particular interest to Israel and *IJHPR*. In addition, now that The Advisory Committee for Strengthening the Public Health System (the "German Committee") has published its long-awaited report, we hope that IJHPR will receive a variety of submissions related to the committee's analyses and recommendations.

We want to take this opportunity to thank all those whose efforts have contributed to the journal's success, including:

- ● The National Institute of Health Policy, the journal's sponsor, which has provided ongoing financial support for the journal
- ● The Institute's leadership team, which has consistently provided us with sage advice and moral support
- ● The journal's editorial board, which has provided vital input at strategic junctures and has also played a significant role in helping us identify appropriate reviewers and commentators
- ● Biomed Central, our publisher, which provides much of the infrastructure that is vital to the journal's smooth operation
- ● The Israeli health policy/health services research community, whose members have authored most of the original research articles (50) and integrative articles (21) that *IJHPR* has published to date
- ● Colleagues from abroad, who have authored most of the 66 *IJHPR* commentaries published to date and, in doing so, highlighted the international significance of numerous studies of Israeli health care
- ● The over 230 reviewers, from Israel and around the world, who have consistently combined rigor and courtesy to provide our authors with first-rate input

In Jewish tradition, the transition from a new endeavor's third year to its fourth year is full of significance. After planting a new tree, it is only in the fourth year that one begins to eat the fruits (which in the fourth year are brought to Jerusalem to be eaten there) [4]. According to one scholar this is because only at that point has the tree, and its fruit, reached an appropriate level of maturity and fullness [5]. In some social and religious circles, a toddler's first haircut takes place at his/her third birthday [6]. In Jewish jurisprudence, only after three years of uncontested possession of a parcel of land, does a presumption of ownership obtain [7, 8], and only after an action has been adhered to for three times, does it take on a presumption of permanence and stability [8].

As editors, we feel that, after 3 years of publication, *IJHPR* is also in the process of moving into a new phase; a more mature phase, with greater stability and a firmer place in the professional literature. We hope that together with you, our authors and readers, we can use this stability as a springboard for further innovation, experimentation and growth.

We hope and believe that the best is yet ahead.

Sincerely,

Bruce and Avi

## Endnote

^a^The "alltime" access statistics are naturally influenced by how long ago a particular article was published. The IJHPR website also provides a capacity to identify, at any point in time, how often various articles have been accessed over the past year or over the past 30 days. Please see http://www.ijhpr.org/mostviewed for the latest statistics.

## Authors’ information

Avi Israeli is the Dr. Julien Rozan Professor of Family Medicine and Health Care at the Hadassah – Hebrew University Medical Center; Director of the Department of Health Policy, Health Care Management and Health Economics, Hebrew University – Hadassah Braun School of Public Health & Community Medicine; Chief Scientist of the Ministry of Health; and co-editor of *IJHPR*.

Bruce Rosen is Director of the Smokler Center for Health Policy Research at the Myers-JDC-Brookdale Institute, as well as co-editor of *IJHPR*.
